# Highly sensitive detection of influenza virus with SERS aptasensor

**DOI:** 10.1371/journal.pone.0216247

**Published:** 2019-04-25

**Authors:** Vladimir I. Kukushkin, Nikita M. Ivanov, Anastasia A. Novoseltseva, Alexandra S. Gambaryan, Igor V. Yaminsky, Alexey M. Kopylov, Elena G. Zavyalova

**Affiliations:** 1 Institute of Solid State Physics RAS, Chernogolovka, Moscow district, Russian Federation; 2 Chemistry Department, Lomonosov Moscow State University, Moscow, Russian Federation; 3 Chumakov Federal Scientific Center for Research and Development of Immune and Biological Products RAS, Moscow, Russian Federation; University of Houston, UNITED STATES

## Abstract

Highly sensitive and rapid technology of surface enhanced Raman scattering (SERS) was applied to create aptasensors for influenza virus detection. SERS achieves 10^6^−10^9^ times signal amplification, yielding excellent sensitivity, whereas aptamers to hemagglutinin provide a specific recognition of the influenza virus. Aptamer RHA0385 was demonstrated to have essentially broad strain-specificity toward both recombinant hemagglutinins and the whole viruses. To achieve high sensitivity, a sandwich of primary aptamers, influenza virus and secondary aptamers was assembled. Primary aptamers were attached to metal particles of a SERS substrate, and influenza viruses were captured and bound with secondary aptamers labelled with Raman-active molecules. The signal was affected by the concentration of both primary and secondary aptamers. The limit of detection was as low as 1 · 10^−4^ hemagglutination units per probe as tested for the H3N2 virus (A/England/42/72). Aptamer-based sensors provided recognition of various influenza viral strains, including H1, H3, and H5 hemagglutinin subtypes. Therefore, the aptasensors could be applied for fast and low-cost strain-independent determination of influenza viruses.

## Introduction

Surface-enhanced Raman scattering (SERS) has been under intense investigation for sensor development in recent years. The main attractive features include the equipment, which is portable and capable of rapid techniques [1], as well as extremely high sensitivity in biological samples [2] and low cost.

For highly sensitive SERS-based sensors complicated substrates are required. Nanostructured metal–dielectric surfaces provide localization of the electromagnetic field in the near-surface zone, resulting in giant enhancement of the signal, typically 10^6^ times [3,4]. A variety of SERS–substrates comprise colloid particles of different sizes and shapes, composite nanoparticles with core–shell structures, and solid-state substrates with intermittent metallic and dielectric layers with both stochastic and periodic structures [5–10]. Silver was shown to be an optimal metal component of the SERS–substrate as it has highly negative real and small positive imaginary components of the permittivity [11,12].

A huge variety of described SERS-based techniques can be organized into two groups: label-free and reporter-based techniques. The latter techniques were shown to be more sensitive and specific for biological samples. Moreover, in this case, the signal was proportional to analyte concentration [2,13].

Molecular recognizing elements (MoREs) play a central role in reporter-based techniques [14]. One of the productive variants is a sandwich-like assay, where primary MoREs are coupled to SERS substrates, whereas secondary MoREs are coupled to SERS-active compounds. The analyte is complexed with both MoREs, providing high concentration of the reporter near the substrate surface [15–20].

Common MoREs, antibodies, have significant disadvantages in sandwich-like SERS techniques, namely non-uniform protein orientation on the surface due to non-specific adsorption on the surface and significant remoteness of the SERS–active compound from the surface, resulting in a drastic decrease in signal intensity [14,21]. Nucleic acid aptamers are more attractive for this application since they can be easily modified in a sequence-specific manner with an anchor for immobilization on the metal surface and a variety of SERS reporters [22–25]. Moreover, typical aptamers are 10-fold smaller than antibodies, which is expected to substantially decrease the distance between reporter and SERS substrate. The overall background of SERS-based sensors is sufficiently elaborated to develop diagnostic tools for different medicinal tasks, including detection of pathogens [26–28].

Influenza viruses are significant human respiratory pathogens that cause both seasonal, endemic infections and periodic, unpredictable pandemics [29]. Highly sensitive and express techniques are required to detect the virus. Today, there are either highly sensitive techniques (enzyme-linked immunosorbent assay, ELISA, or polymerase chain reaction, PCR) or rapid techniques (immune–chromatography strips). The gap between sensitive and express techniques comprises 10^2^ to 10^4^-fold differences in the limit of detection and depends on viral strain [30–33]. SERS-based techniques were shown to have nearly the same limit of detection as ELISA in the case of hepatitis B antigen detection [15] and the PCR technique in the case of the whole virus of porcine circovirus [34]. Thus, SERS could be a good approach for combining both sensitivities and rapid detection in one pot.

Current SERS-based techniques for influenza virus detection have low sensitivity comparing to ELISA or PCR [35–39]. There are only a few studies dedicated to SERS-based aptasensors for detection of viruses [37,38], although this novel area is promising due to combination of aptamers’ specificity and signal amplification provided by SERS.

The present study aimed to develop SERS-based aptasensor for highly sensitive and express detection of different strains of influenza virus in biological fluids.

## Material and methods

### Reagents

Phosphate buffered saline (PBS) tablets were from Ecoservice, Russia. Glutaric aldehyde, NaN_3_, KCl, NaCl, dithiothreitol (DTT), dimethyl sulfoxide (DMSO), 3,3',5,5'-tetramethylbenzidine, Tween-20 and other chemicals were from Helicon, Russia. The 3% H_2_O_2_ solution was from Rosbio, Russia. Recombinant hemagglutinins H1, H3, H5, H7, and H9 (Abcam, USA), fetuin from fetal bovine serum (Sigma, USA), and streptavidin horseradish peroxidase conjugate (Str-HRP) (Lot 16731090, RPN1231, GE Healthcare, USA) were used.

Compositions of buffers used were as follows. PBS: pH 7.4, 8 mM Na_2_HPO_4_, 136 mM NaCl, 1.5 mM KH_2_PO_4_, 2.7 mM KCl. PBS+K: PBS with additional 16 mM KCl. PBS+K+T: PBS+K supplemented with 1/10000 (v/v) Tween-20. PBST: PBS supplemented with 1/2000 (v/v) Tween-20. Str-HRP working buffer: PBS + 0.05% (w/v) bovine serum albumin + 1/10000 (v/v) Tween-20. All solutions were prepared in deionized water (MilliQ).

DNA oligonucleotides were chemically synthesized as follows: unmodified aptamer, RHA0385 (5’–ttggggttattttgggagggcgggggtt–3’) and control sequence, TBA (5’–ggttggtgtggttgg –3’) were from Evrogen, Russia; thiolated, RHA0385–SH (5’–HS–(CH_2_)_6_– ttggggttattttgggagggcgggggtt–3’) and biotinylated, RHA0385–Biotin (5’–Biotin–ttggggttattttgggagggcgggggtt–3’) aptamers were from Syntol, Russia; and fluorescently labelled aptamers, RHA0385–Cy3 (5’–Cyanine 3–ttggggttattttgggagggcgggggtt–3’), RHA0385–BDP FL (5’–Bodipy FL–ttggggttattttgggagggcgggggtt–3’) were from Syntol, Russia and Lumiprobe, Russia, respectively.

Influenza viruses (IV) and allantoic fluid were provided by Chumakov Federal Scientific Center for Research and Development of Immune and Biological Products of Russian Academy of Sciences. Viruses: A/Puerto-Rico/8/1934 (H1N1), A/New Caledonia/10/1999 (H1N1), A/England/42/1972 (H3N2), A/Mississippi/1/1985 (H3N2), A/Aichi/2/1968 (H3N2), A/Buryatia/652/1988 (H3N8), A/duck/Buryatia/664/1988 (H3N2), A/Buryatia/2408/2001 (H4N6), A/chiken/Kurgan/3654-at/2005 (H5N1), A/Vietnam/1203/2004-PR8/CDC-R (H5N1), A/duck/Moscow/4182/2010 (H5N3), A/tern/South Africa/1/1961 (H5N3), A/mallard/Sweden/91/2002 (H7N9), A/Primorie/3631/2002 (H9N2), A/duck/Primorie/3691/2002 (H12N2). Paramyxovirus was used as a negative control.

Virus stocks were propagated in the allantoic cavity of 10-day-old embryonated SPF chicken eggs (CE). CE were incubated at 37°C, cooled at 4°C 48 h post infection and harvested 16 h latter. The study design was approved by the Ethics Committee of the Chumakov Institute of Poliomyelitis and Viral Encephalitides, Moscow, Russia (Approval #4 from 2 December 2014). Viruses were inactivated via the addition of 0.05% (v/v) glutaric aldehyde, preserved via the addition of 0.03% (w/v) NaN_3_ and stored at +4°C.

### Hemagglutination tests for IV characterization

These were carried out following the standard protocol [40]. V-bottom 96-well plates for hemagglutination tests were from Greiner, Austria. Virus loads in viral particle per ml, VP/mL, were estimated from hemagglutination units, HAU/mL, based on correlations published earlier [41].

### Preformation of aptamers

Solutions of 1 μM RHA0385 and 1 μM TBA were prepared in PBS+K; 1 μM RHA0385-SH was prepared in 10 mM KCl; and 4 μM RHA0385–Biotin, 9 μM RHA0385–Cy3 and 9 μM RHA0385–BDP FL were prepared in PBS+K buffer. The solutions were heated to 95°C for 5 min and cooled down to room temperature (r.t.) in air.

### Surface plasmon resonance

Experiments were conducted on a GLM chip of the ProteOn XPR36 system (Bio-Rad, USA) at 25°C. Recombinant hemagglutinins H1, H3, H5, H7, and H9 were immobilized by amine coupling from 10 μg/mL protein solutions in 10 mM acetate buffer at pH 5.0. Analyte solutions were 25, 50, 100, and 200 nM RHA0385 aptamer in PBS+K. A 100 nM solution of TBA in PBS+K was used as a negative control. The signal from the TBA binding did not exceed the signal from PBS+K. At least two sensorgrams without spikes were obtained for each aptamer–hemagglutinin complex. Solutions of 300 mM NaCl and 0.01% Tween-20 in PBS were injected after measurements to regenerate proteins. The chip surface without immobilized protein was used as a reference. Values of kinetic constants of complex association (k_on_) and dissociation (k_off_) were determined using exponential approximations of complex association and dissociation portions of the sensorgrams, correspondingly. Apparent dissociation constants aK_D_ were calculated from the equation aK_D_ = k_on_/k_off_.

### Enzyme-linked aptamer assay (ELAA)

About 100 μL of a 10 μM fetuin solution in 140 mM NaCl was adsorbed over 24 h at r.t. to wells of a polystyrene 96-well plate for ELISA (Medpolimer, Russia). The solution was removed, and the wells were washed three times with 200 μL of distilled water. Solutions of IV were diluted to 128 HAU with 140 mM NaCl. About 50 μL of the suspensions were added to each well of the fetuin plate. After incubation for 24 h. at r.t. the wells were washed 5 times with 100 μL PBST. About 50 μL of preformed biotinylated aptamer (with 1/10000 (v/v) Tween-20 added after preformation) in PBS+K+T were added with serial 2-fold dilutions in the concentration range of 0–1000 nM. After 1 h incubation at r.t. the wells were washed 5 times with 100 μL PBST. About 50 μL of 1/1000 (v/v) solution of streptavidin horseradish peroxidase conjugate in Str-HRP working buffer were added. After 1 h incubation at r.t. the wells were washed 5 times with 100 μl PBST. 50 μl of substrate solution (0.05 mg/ml 3,3',5,5'-tetramethylbenzidine, 0.033% H_2_O_2_ in 0.05 M acetate buffer pH 4.5) were added; the peroxidase reaction was carried out for 30 min at r.t. and stopped by the addition of 100 μL 5% (v/v) H_2_SO_4_. Absorption at a wavelength of 450 nm was measured with a TECAN Spark 10M microplate reader. Curves were processed using Origin 13 software and apparent dissociation constants were determined from approximation with the exponential decay function.

### SERS substrate preparation and SERS equipment

Two nanostructured silver zones were deposed on a silicon plate covered with a 300 nm thick layer of SiO_2_ using the thin film deposition system NANO 38 (Kurt J. Lesker Company, USA). As a result, the zones were covered with a 6 nm thick layer of silver granules with a mean planar size of 20 nm. The surface coverage was studied with scanning electron microscopy (SEM) using a JEOL JSM-7001F microscope (JEOL Ltd., Japan). One of the zones was used as the experimental zone, and another zone was the control (S1 Fig). A Raman spectrometer (EnSpectr SERS R532, Enhanced Spectrometry, USA) with an excitation wavelength of 532 nm was used for instant and automatic measurement of the SERS signal from reporter molecules. Intensities were measured as the integral over surface area. The spectrometer had a resolution of 8 cm^–1^ throughout a spectral range of 120–3400 cm^–1^. The effective emissive power of the laser was 30 mW, and the diameter of the beam was 2 mm. The wide beam provided low density of irradiance, which prevented the dyes from burning out and registered the mean integral intensity over a large area from many reporter molecules. The photoluminescence signal was subtracted automatically using the software for the spectrometer.

### SERS-based aptasensor testing

SERS substrates were subsequently placed in wells of a non-sorbing 96-well plate (Greiner, Austria) with the following reagents:

1) Immobilization of primary aptamers. 20 nM RHA0385-SH in water (or 0.2–1500 nM for concentration dependence). Time of incubation was 5 min.

2) Surface ‘blocking’ with 1 μM DTT. Time of incubation was 2 min.

3a) Virus binding to the experimental zone. IV dilutions in PBS+K with concentrations in the range of 6.4·10^−5^ to 6.4 HAU/mL. Time of incubation was 2 min.

3b) Non-specific binding of allantoic fluid components to the control zone. Allantoic fluid dilutions prepared in PBS+K. Time of incubation was 2 min.

4) Staining with secondary aptamers. 9 μM RHA0385-Cy3 or 9 μM RHA0385-BDP FL in PBS+K (or 0.037–9 μM for concentration dependence). Time of incubation was 2 min.

5) Removal of salts with MilliQ water. Time of incubation was 1 min.

The total duration was 12 min.

Then the substrates were placed horizontally, dried in air, and subjected to SERS signal measurement.

### Atomic force microscopy scanning

This was performed under ambient conditions using a FemtoScan atomic force microscope (Advanced Technologies Center, Russia) [42] in contact mode (applied force approximately 1 nN) using high-resolution silicon cantilevers and mechanical rigidity of 0.1 N/m (fpC01, Lukin Institute of Physical Problems, Russia). Signal processing and imaging was performed using FemtoScan Online software [43]. SERS substrates were scanned just after SERS experiments and drying in air at r.t. for 2 h.

## Results and discussion

### Estimation of aptamer specificity to influenza viruses

**Fig 1:**
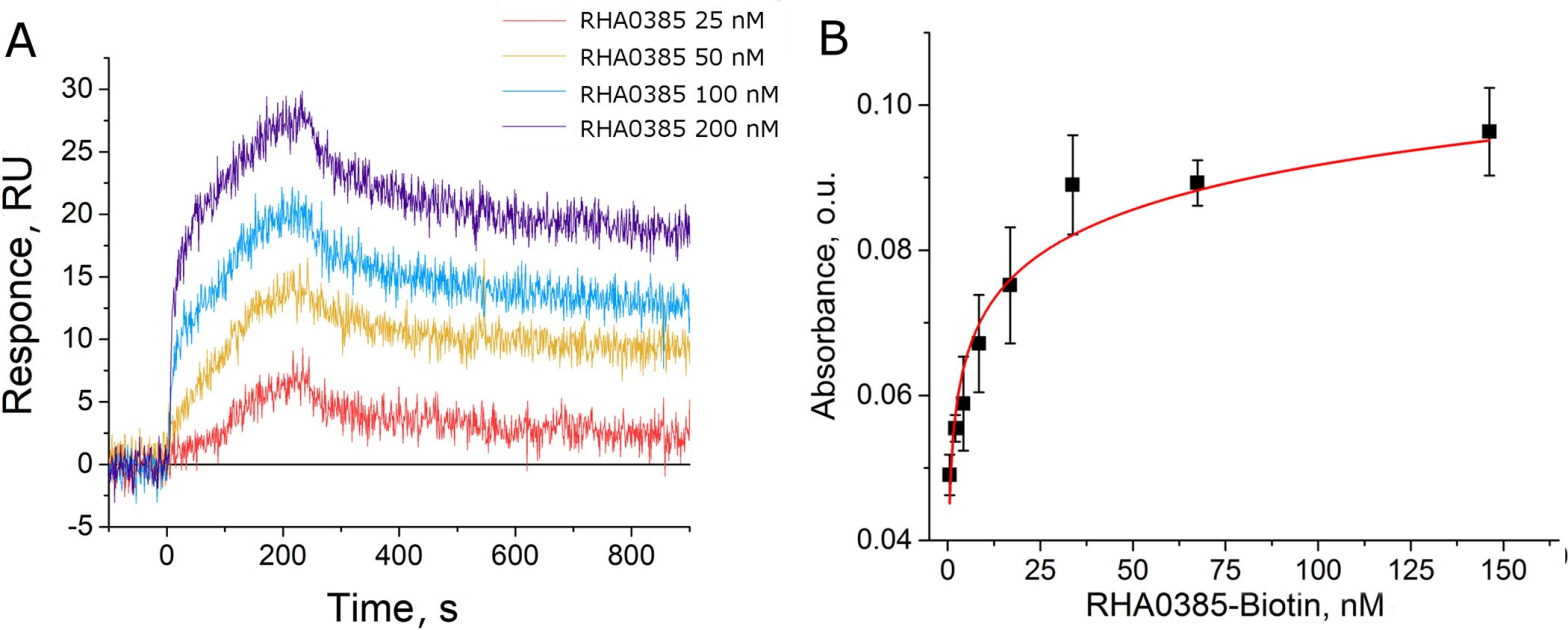
Binding experiments. (A) SPR sensorgrams of binding RHA0385 to immobilized recombinant HA of H3 subtype and (B) an ELAA curve for biotinylated aptamer binding to the whole IV A/Aichi/2/1968 (H3N2).

Aptamer RHA0385 was selected by Shiratori *et al*. [44] to recombinant hemagglutinin from H5N1 influenza strain. The aptamer was shown to bind both recombinant hemagglutinins (HA) and the whole influenza viruses (IV) of H1N1, H3N2, and H5N1 strains [44]; however, affinity constants were not determined. Here, we extended the set of recombinant HA subtypes up to 5 species and determined the aptamer affinity using the surface plasmon resonance technique (SPR) (Fig 1). The apparent dissociation constants of aptamer–HA complexes are in the range of 7–14 nM (Table 1) exhibiting no specificity to the HA subtype.

**Table 1:** Broad strain-specificity of the RHA0385 aptamer.

HA subtype	Recombinant HA	The whole viruses	
	aK_D_, nM	Influenza A virus strains	‘+’–binding, ‘-’–no binding, values—aK_D_, nM
**H1**	7 ± 2	Puerto-Rico/8/1934 (H1N1)	13 ± 2
		New Caledonia/20/1999 (H1N1)	42 ± 14
**H3**	12 ± 3	Mississippi/1/1985 (H3N2)	+
		duck/Buryatia/664/1988 (H3N2)	+
		Aichi/2/1968 (H3N2)	17 ± 5
		England/42/1972 (H3N2)	40 ± 5
**H5**	13 ± 3	chicken/Kurgan/3654at/2005 (H5N1)	+
		Vietnam/1203/2004(H5N1)-PR8/CDC-R	34 ± 8
		tern/South Africa/1/1961 (H5N3)	200 ± 50
**H7**	12 ± 4	mallard/Sweden/91/2002 (H7N9)	13± 4
**H9**	14 ± 4	Primorie/3631/2002 (H9N2)	16 ± 3
**H12**	n.d.	duck/Primorie/3691/2002 (H12N2)	> 200
**-**	-	Paramyxovirus (negative control)	No binding
		(Influenza B) B/Victoria/2/1987	

Apparent dissociation constants (aK_D_) of the complex of the aptamer with recombinant HA were determined with SPR. The capacity for whole virus binding with the aptamer was assessed by ELAA; aK_D_ values were determined for several IV strains. Standard deviations are provided. n.d., not determined.

These results were supported with binding to the whole viruses assessed by the ELAA technique (aptamer-based analogue of ELISA). In this sandwich-like technique, viruses were adsorbed on highly glycosylated protein (fetuin), secondary binding agent, biotinylated aptamers bound to the virus, and then biotin quantity was estimated enzymatically using streptavidin-peroxidase conjugate. The high quality of some curves allowed for the calculation of apparent dissociation constants (Table 1, Fig 1). The range of the constants for IV is considerably broader than that for recombinant proteins, namely 17–200 nM versus 7–14 nM, correspondingly. Notably, the lack of binding was observed only for the viruses with H9 and H12 subtypes, which are maximally divergent from other strains studied [45]. These subtypes do not cause influenza epidemics in humans [46]. The results of binding assays suggest RHA0385 as a promising MoRE for strain-unspecific detection of IVs circulating in humans.

### Design and tuning of SERS-based aptasensor

**Fig 2:**
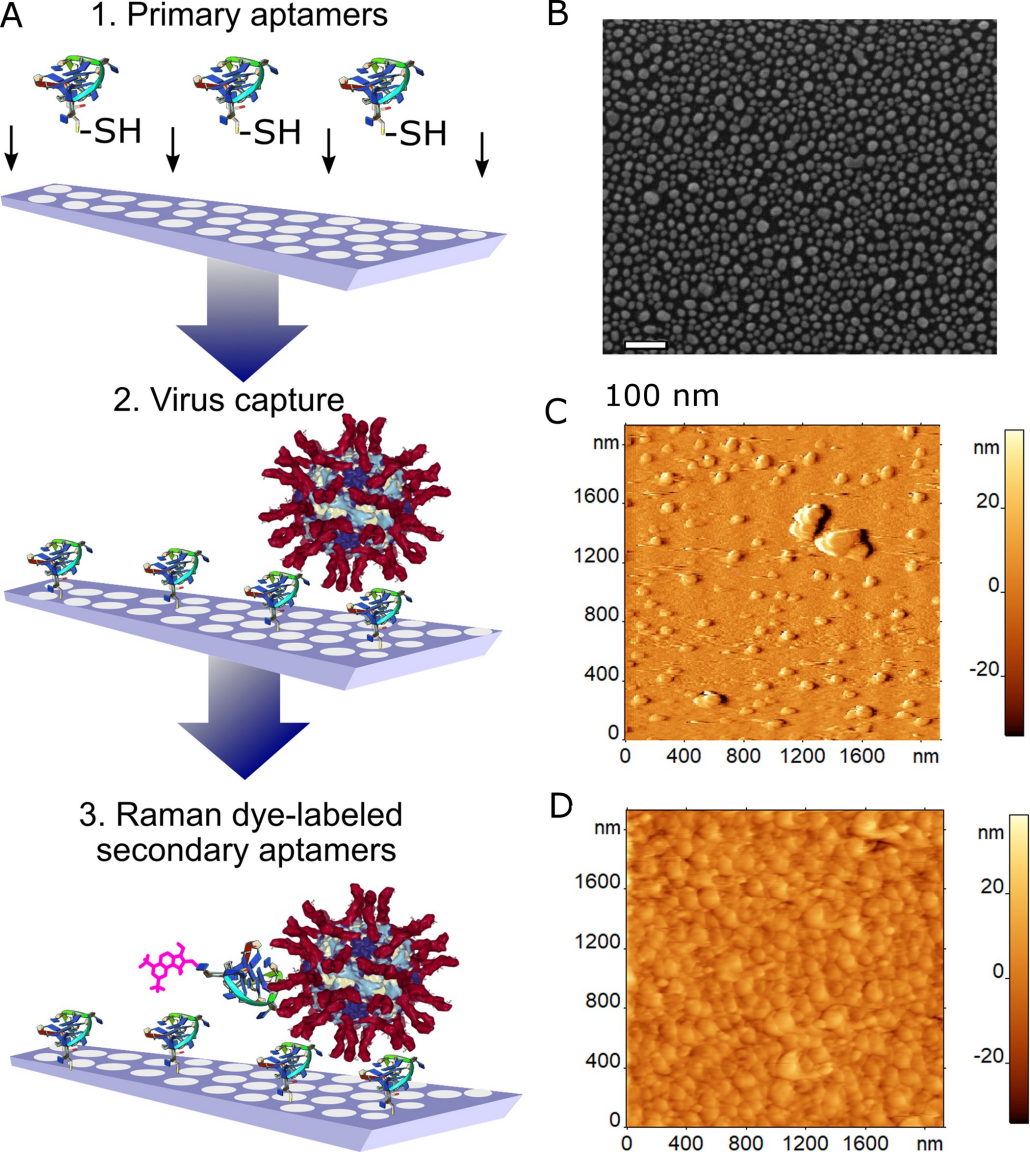
Design of aptasensor for influenza virus. (A) Scheme of sandwich-like aptasensor for IV detection: 1) primary aptamer is immobilized onto Ag nanoparticles, 2) IV is captured with primary aptamers, 3) secondary aptamers interact with IV, providing the SERS signal. (B) SEM image of the empty substrate. (C) AFM image of the control zone with adsorbed components of allantoic fluid. (D) AFM image of the experimental zone with a dense layer of virus particles adsorbed from the sample with 0.64 HAU/mL concentration.

Over the course of the research, SERS substrates (Fig 2B) were optimized for the used wavelength of laser radiation of the spectrometer (532 nm), which provides high sensitivity of Raman scattering detection compared to low-frequency laser excitation. Simple and affordable nanoisland SERS substrates are reproducible in manufacturing and relatively low cost. They are stable to chemical processes and have a plasmon resonance near 500 nm. Previously, the authors have shown that this type of nanoisland SERS substrates have a higher enhancement factor than complex nanolithographic structures at the laser excitation wavelength of 532 nm [12].

Sandwich-like techniques are expected to provide high sensitivity and selectivity. In the aptasensor, the primary aptamers are immobilized onto Ag nanoparticles via a thiol group. IV particles are captured with primary aptamers, whereas secondary aptamers with a SERS reporter provide the analytical signal (Fig 2A). Since viruses were obtained in allantoic fluid, the allantoic fluid with no IV was used as a control. Both fluids were equally diluted with the same buffer. In our design, both control and experimental SERS zones were placed onto the same substrate.

As seen from atomic force microscopy (AFM), the control zone trapped some sporadic biomolecules from the allantoic fluid that could be attributed to biomolecule aggregates (Fig 2C). The object height was below 10 nm. On the contrary, the experimental zone was covered with a dense layer composed of IV particles (Fig 2D). The particles were packed so tightly that the substrate surface could not be achieved with the cantilever without damaging the layer. These images confirmed the efficient trapping of IV with immobilized primary aptamers.

**Fig 3:**
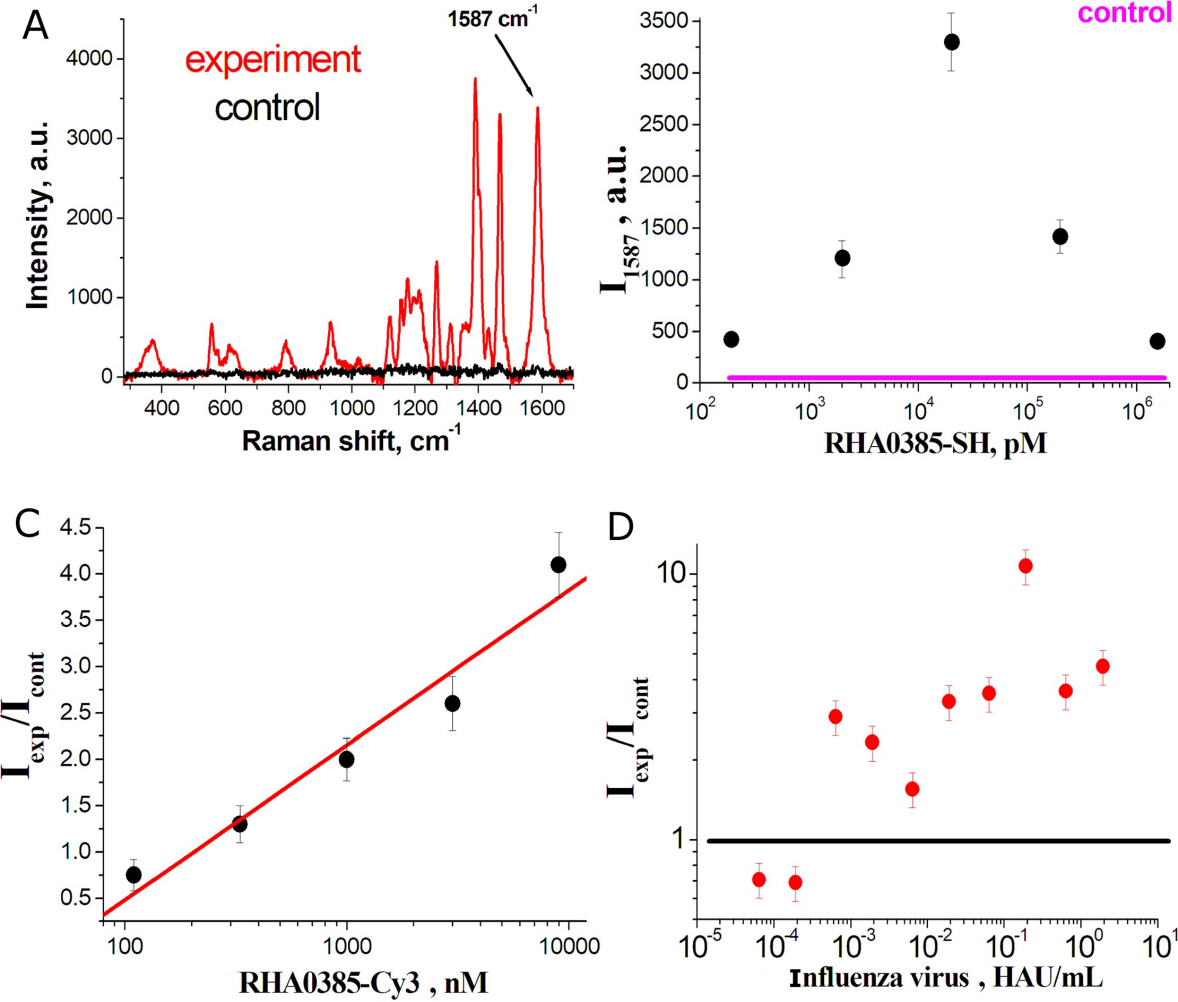
Optimization of SERS-based aptasensor. (A) SERS spectrum of the Cy3-labeled sandwich-like assay. The intensity of marked peak was taken as analytical signal. The dependencies of the analytical signal on (B) the concentration of primary aptamers (viral load was 6.4·10^−2^ HAU/mL), (C) secondary aptamers (viral load was 6.4·10^−2^ HAU/mL, coefficient of determination for the logarithmic dependence was R^2^ = 0.96), and (D) viral particles of A/England/42/1972 (H3N2).

Specific detection of captured IV was possible using Cy3-labeled RHA0385 as secondary aptamers (Fig 3). The typical Raman spectra of the sandwich corresponds to the spectrum of the Cy3 dye alone (Fig 3A and S2 Fig). The intensity of the peak at 1587 cm^–1^ was chosen as the analytical signal for further estimations.

The analytical signal depended on a variety of parameters. We tested protein, polymer and low-molecular blocking substances. Protein (human serum albumin, 0.1–0.01%) provided very low signal in the control zones (diluted allantoic fluid) but the signal in experimental zone (diluted influenza virus) was the lowest compared to other blockers. Polyethylene glycol (0.1–0.01%) provided high signals in both zones giving signal ratio near 1.0 (signal ratio is a ratio of signal in experimental zone to signal in control zone; it is to be >1 in case of influenza virus capture and = 1 in case of virus absence). DTT is low-molecular compound with thiol groups; it binds readily to silver nanoparticles. In our experiments DTT was the most efficient blocker providing signal ratio >1 in wide range of concentrations of other constituents.

The signal depended strongly on the concentration of the primary and secondary aptamers (Fig 3B). The dependence on concentration of the primary aptamers was bell-shaped with a maximum signal at ~10 nM that reflects the necessity of sub-monolayer adsorption of the aptamer. On the contrary, the dependence on concentration of secondary aptamers was monotonous and can be linearly approximated; the signal increased with the increase in secondary aptamer content (Fig 3C).

The dependence of the signal on the virus concentration had a more complex shape (Fig 3D). We speculate that this non-monotonous dependence could be explained with the following. The distance between Raman-active label and enhancing surface determines the signal intensity. When IV particles form a dense layer (Fig 2D), the reporter molecules are remoted from the surface, and the signal decreases. This phenomenon gives the complex shape of the dependence (Fig 3D).

Despite the non-monotonous variations in the signal, the analytic signal was 1.5- to 10-fold higher in the experimental zone as compared to the control zone for a wide range of virus concentrations (from 5·10^−4^ to 2 HAU/mL). The limit of detection (LOD) was estimated from the lowest virus content with signal ratio > 1.0. Since the sample volume was 250 μL, the LOD was 1.3·10^−4^ HAU per sample or 2·10^4^ virus particles per sample (ratio between HAU/mL and VP/mL units was taken from Kramberger *et al*. [41].

**Fig 4:**
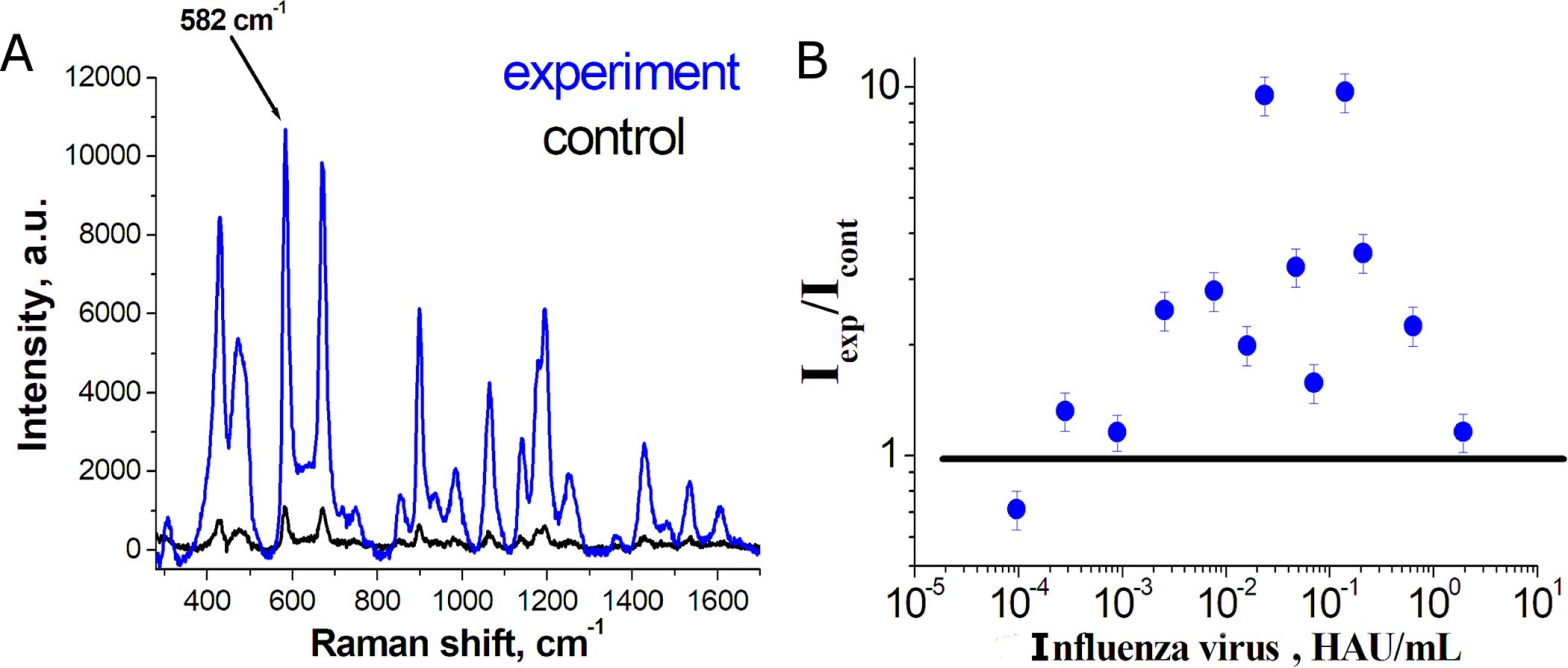
Aptasensor with BDP FL as SERS reporter. (A) SERS spectrum of the BDP FL dye in the sandwich-like aptasensor. The intensity at 582 cm^–1^ was taken as the analytical signal. (B) The dependency of the analytical signal on the concentration of viral particles of A/England/42/1972 (H3N2).

The Cy3 dye exerts marked fluorescence in the range of the Raman spectra of the dye. One more SERS reporter, Bodipy FL, was tested. Bodipy FL (BDP FL) exerts no fluorescence in this range, and typical intensities of the signal are higher when BDP FL-labeled RHA0385 is used as the secondary aptamer (Fig 4). The dependence of analytical signal (intensity of the peak at 582 cm^–1^) on virus concentration was non-monotonous; it could be approximated roughly as bell-shaped. The analytical signal was higher in the experimental zone as compared to the control zone for a wide range of virus concentrations (from 2.5·10^−4^ to 1.3 HAU/mL). The LOD was estimated to be 7·10^−5^ HAU per sample or 1·10^4^ virus particles per sample. This result is quite similar to the results with Cy3-labeled RHA0385.

Next, we estimated reproducibility of SERS signal for the same sample. The main reasons of fluctuations are: 1) Non-reproducible distribution of shapes and sizes of metal nanoparticles on the substrate surface. We used technology of vacuum thermal spraying with automatically controlled and very low metal spraying rate of 0.1 A/s, which allows creating a batch of reproducible SERS-structures with a homogeneous SERS-gain in one deposition cycle. 2) The heterogeneity of the distribution of substances involved in the assembly of a full sandwich on the surface. This difficulty was diminished using a) thiol-modified aptamers that are expected to be oriented more uniformly than adsorbed proteins and b) Raman spectrometer with a wide laser beam, which in one measurement averages the signal from almost the entire surface area of the SERS substrate (4 mm^2^) and thereby minimizes the measurement error compared to microscopic Raman systems in which the signal is recorded from sites of the order of 10 μm^2^. 3) Decay in SERS signal intensity during irradiation by a powerful focused laser beam. Enspectr Raman spectrometer had a wide laser beam; the radiation power density is low allowing usage of large exposure times by the laser beam.

For reproducibility test, optimal concentrations of primary aptamers (20 nM), IV (0.064 HAU/mL), and secondary aptamers labeled with Cy3 (9 μM) were used. The reproducibility of the measurement was tested for IV A/England/42/1972 (H3N2). Seven measurements at 3 different lots of SERS substrates gave a relative intensity from 2.56 to 3.93 with a mean value of 3.1±0.5 (relative measurement error was 16%).

The same conditions, namely 20 nM of primary aptamers, 0.064 HAU/mL of IV, and 9 μM of secondary aptamers labeled with Cy3 were used to study strain specificity of the aptasensor. The full list of influenza A viruses tested includes H1N1, H3N2, H3N8, H4N6, H5N1, H5N3, H9N2 and H12N2 strains (Table 2). The aptasensor detected readily all these strains, which means that the aptasensor is suitable for seasonal (H3N2), pandemic (H1N1), and rare outbreaks (H5N1 and others) of influenza viruses.

**Table 2:** Testing strain specificity of the aptasensor with Cy3-labeled secondary aptamers.

Influenza virus A strains	IV subtype	Relative SERS signal
**New Caledonia/20/1999**	H1N1	1.7±0.4
**A/England/42/1972**	H3N2	3.0±0.5
**A/Mississippi/1/1985**	H3N2	4.3±0.6
**A/Aichi/2/1968**	H3N2	4.1±0.6
**A/Buryatia/652/1988**	H3N8	2.4±0.5
**A/Buryatia/2408/2001**	H4N6	6.9±0.6
**Vietnam/1203/2004-PR8/CDC-R**	H5N1	4.8±0.5
**duck/Moscow/4182/2010**	H5N3	2.9±0.5
**A/South Africa/1/1961**	H5N3	6.5±0.6
**A/Primorie/3/1982**	H9N2	2.4±0.3
**A/Primorie/3691/2002**	H12N2	5.0±0.5

Ten IV were chosen with H1, H3, and H5 hemagglutinin subtypes. Values of relative SERS signal >1 indicate detection of viral particles.

### Comparison with other techniques for influenza virus detection

The LOD of SERS-based aptasensor was estimated to be 7·10−5–1.3·10^−4^ HAU per sample or 1·104–2·10^4^ virus particles per sample. It is 10^1^ to 10^3^ times lower than most values published for other aptamer-based approaches for the virus, i.e. 10^−1^ to 10^−3^ HAU [47–50]. The only aptamer-based technique with the lowest LOD was described by Kiilerich-Pedersen *et al*. [51]. The LOD was as low as 2 pfu/probe, which is ≈ 2·10^2^ virus particles per sample (recalculation was made using data from Kramberger *et al*. [41]. The technique detected subtle changes in the impedance of the microelectrode in a microfluidic channel.

As for non-aptamer SERS-based techniques, detection of influenza genome via complementary DNA probe was shown to have no advantages in terms of LOD having sensitivity 2.7 attomole of RNA per probe (nearly 1.5·10^5^ virus particles per sample) [52]. SERS-based lateral flow immunoassay had 10-fold higher LOD as tested for H7N9 influenza virus [53].

As compared to common laboratory techniques for IV determination, our aptasensor had a 100-fold lower LOD than immunochromatographic techniques, conceding only to the PCR techniques only, which has the LOD in the range of 10^2^ to 10^3^ virus particles [30]. Two key features allowed achieving the high sensitivity in our assay. The first feature was excitation with the 532 nm laser that corresponds to the shortwave range. The second feature was optimization of the peak of plasmon absorption of SERS substrates so that it is close to excitation wavelength. The optimized SERS substrates were as efficient as metal periodic clusters at 532 nm excitation wavelength [12] but are much more easy and cheap in production.

Further efforts could be implemented to enhance the sensitivity. The sensitivity of SERS-based IV detection could be enhanced using nanoparticles instead of a SERS reporter. Proof of the principle was demonstrated for an antibody-based microfluidic sensor with SERS detection, where the LOD was as low as 74 pg/mL [13], which corresponds to 2.5·10^2^ VP/mL if using 180 MDa as the mass of a virus particle. Although the LOD of our technique was low but not the lowest one; the technique had obvious advantages as it does not requires antibodies, microfluidics, or nanoparticles. The total time for sandwich assay and measurement of a single sample does not exceed 20 minutes. The cost of consumables for the single analysis is low being less than ten dollars.

## Conclusions

The SERS-based aptasensor with broad strain specificity to influenza viruses was developed. The limit of detection was at the level of 10^4^ virus particles per sample or 10^−4^ HAU per sample, which is substantially lower than the values for other rapid techniques commonly used for IV detection. H1, H3, and H5 IV subtypes can be readily detected using the RHA0385 aptamer as recognizing element. The technique requires only 12 min for full analysis and uses low cost reagents, making it attractive for further implementation.

## Supporting information

S1 Fig Design of SERS substrate. Substrate length is 20 mm and width is 4 mm.
(TIFF)

S2 Fig SERS spectra of fluorescent dyes Cy3 and BDP FL. (TIF)
