# Supplementary figures and images for: Highly sensitive detection of influenza virus with SERS aptasensor
Source: PLoS One. 2019 Apr 25;14(4):e0216247. doi: 10.1371/journal.pone.0216247 (PMC6483365; doi:10.1371/journal.pone.0216247)

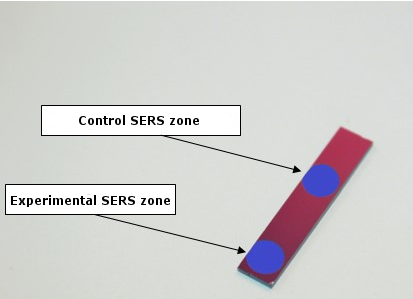

Supplement: S1 Fig — Substrate length is 20 mm and width is 4 mm. (TIFF) [file pone.0216247.s001.tiff]
